# Establishment and evaluation of module-based immune-associated gene signature to predict overall survival in patients of colon adenocarcinoma

**DOI:** 10.1186/s12929-022-00867-2

**Published:** 2022-10-14

**Authors:** Jing Lu, Francesco Annunziata, Dovydas Sirvinskas, Omid Omrani, Huahui Li, Seyed Mohammad Mahdi Rasa, Anna Krepelova, Lisa Adam, Francesco Neri

**Affiliations:** 1 Leibniz Institute on Aging, Fritz Lipmann Institute (FLI), Jena, Germany; 2 Present Address: Life Sciences and Systems Biology Department, University of Torino, MBC, via Nizza 52, 10126 Turin, Italy

**Keywords:** Colon adenocarcinoma, Immune tumor microenvironment, Prognosis, Risk model, Cancer inflammation, NCOA7, Immunoglobulin

## Abstract

**Background:**

Patients with colon adenocarcinoma (COAD) exhibit significant heterogeneity in overall survival. The current tumor-node-metastasis staging system is insufficient to provide a precise prediction for prognosis. Identification and evaluation of new risk models by using big cancer data may provide a good way to identify prognosis-related signature.

**Methods:**

We integrated different datasets and applied bioinformatic and statistical methods to construct a robust immune-associated risk model for COAD prognosis. Furthermore, a nomogram was constructed based on the gene signature and clinicopathological features to improve risk stratification and quantify risk assessment for individual patients.

**Results:**

The immune-associated risk model discriminated high-risk patients in our investigated and validated cohorts. Survival analyses demonstrated that our gene signature served as an independent risk factor for overall survival and the nomogram exhibited high accuracy. Functional analysis interpreted the correlation between our risk model and its role in prognosis by classifying groups with different immune activities. Remarkably, patients in the low-risk group showed higher immune activity, while those in the high-risk group displayed a lower immune activity.

**Conclusions:**

Our study provides a novel tool that may contribute to the optimization of risk stratification for survival and personalized management of COAD.

**Graphical Abstract:**

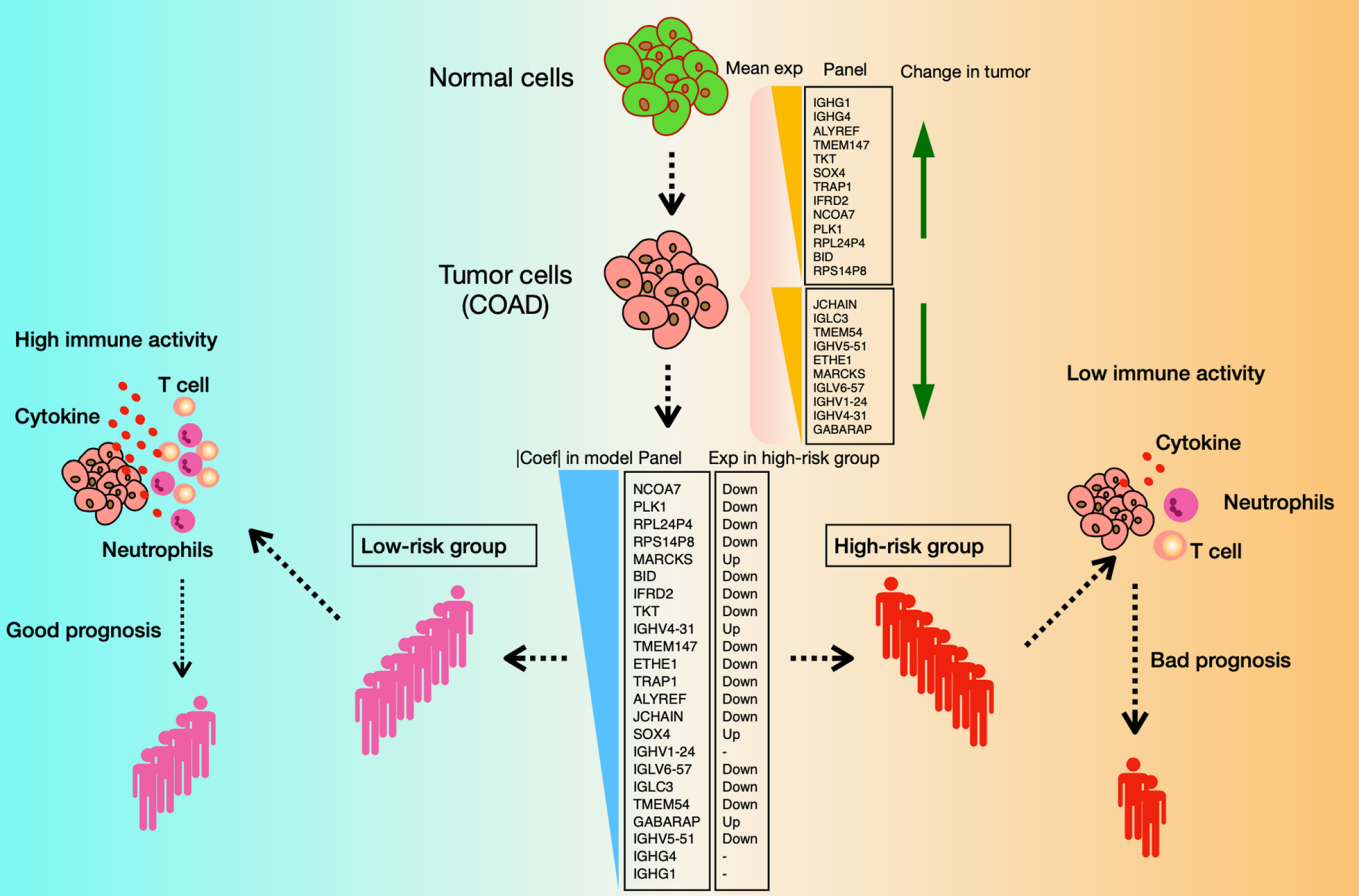

**Supplementary Information:**

The online version contains supplementary material available at 10.1186/s12929-022-00867-2.

## Introduction

Colorectal cancer (CRC) is the second leading cause of cancer death worldwide [1]. Colon adenocarcinoma (COAD) is the most common subtype of CRC [2]. Despite the advancements in earlier diagnosis and treatment over the past decades, the 5-year survival rate of CRC patients remains unsatisfactory [3]. The current prognostic model still relies on conventional clinical predictors such as age, gender, as well as tumor-node-metastasis (TNM) staging [4]. This model results in an inaccurate prognosis due to the high heterogeneity of CRC [5]. Thus, the establishment and application of novel signatures or biomarkers for predicting the survival of CRC patients or instructing the therapy strategy are of great importance in this field. With the development of next generation sequencing (NGS), the high-throughput technology made it possible to screen significant signatures for prognosis in a large scale and improve disease diagnosis, prognosis and treatment.

Tumor microenvironment (TME) is emerging to be related to prognosis in CRC [6]. The changes of TME are considered to be the complex result of multiple variables [6]. Still, the immune interaction was thought to play an important role in this process [7]. Notably, the infiltration of immune cells into CRC tumors was reported to be firmly associated with disease progression and patient survival [7]. Immune therapy for CRC patients was also taken in account as an emerging effective therapy strategy [8]. During the process from tumorigenesis to treatment, involved immune cells, as a complex and multi-faceted role in cancer, take part in suppressing tumor initiation and progression as well as promoting proliferation, infiltration and metastasis [8]. The immune activity involved in tumorigenesis causes the transcriptome changes in tumor cells, which makes it possible to develop an immune-associated signature that effectively responds to clinical outcomes [9]. In this study, we established a risk model comprising 23 genes based on the modules identified from weighted correlation network analysis (WGCNA). The predictive power of the immune signature was identified by the stratification of risk score at the transcriptome level. The prognosis effect of the genes is validated in a large independent cohort. Functional enrichment and immune-activity deconvolution showed immune signatures’ change in risk score-stratified groups.

## Methods

### Schematic diagram of this study design

**Fig. 1:**
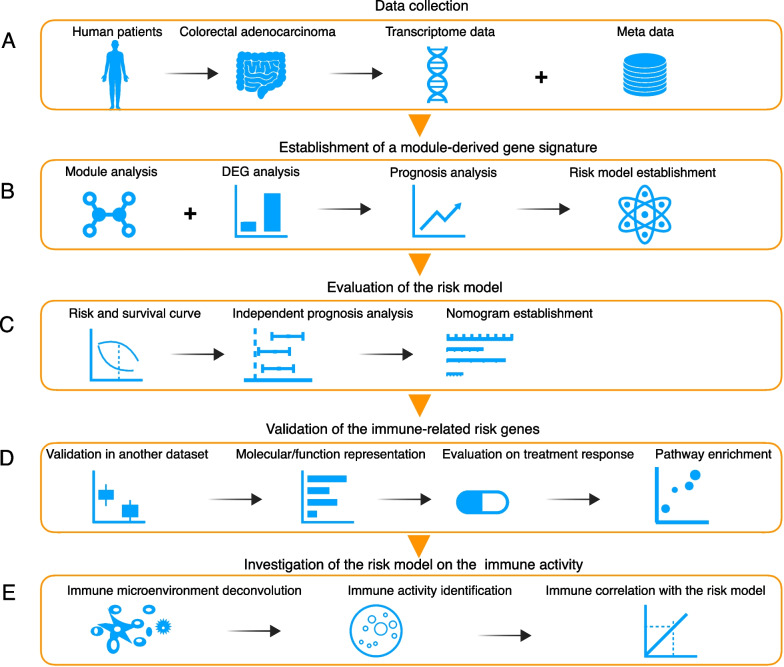
Schematic diagram of the study design. **A** Data of human samples of colorectal adenocarcinoma were obtained from public databases (see Additional file 1: Table S1 and Supplementary Methods). **B** A risk model is established by integrating module analysis and Cox regression. **C** The risk model is evaluated by independent prognosis analysis and a nomo decision tree is established by integrating risk score and other clinical parameters. **D** The risk model is validated in another independent large cohort of colon cancer; molecular classification, therapy evaluation and pathway enrichment analysis of the risk genes is investigated. **E** Tumor microenvironment immune activity is correlated with the risk model

In this study, to identify and investigate a risk model which can help improve the prognosis in colon cancer, we collected data on human patients with colorectal adenocarcinoma (COAD) (Fig. 1A), which is categorized as transcriptome, clinical part and hallmark gene datasets (Additional file 1: Table S1). Correlation modules are identified by weighted correlation network analysis (WGCNA). Differentially expressed genes (DEGs) (adjusted p < 0.05, | log2(fold change) |> 1) were identified among genes of survival-correlated modules. Univariant Cox analysis was performed to predict the prognosis-related module-derived DEGs. The risk model was established by applying stepwise regression to multivariant Cox model and 23 genes (from 174 candidate prognosis-related module-derived DEGs) were selected with coefficient defined for each target gene (Fig. 1B). A risk score was then calculated by summing up the multiplication of gene expression level and its coefficient. Risk assessment and prognosis analysis were performed to evaluate the risk score in predicting survival in COAD patients as an independent parameter. In addition, a comprehensive decision tree of nomogram was constructed based on the risk score and other clinicopathological parameters to improve risk stratification and assessment for individual patients (Fig. 1C). Then, further validation of the risk model was performed in another large independent cohort of 562 samples of colon cancer. Evaluation on the molecular classification, treatment response, pathway enrichment and function representation on the risk model were also applied to provide a deep insight into these risk model genes (Fig. 1D). Risk model genes showed a robust and putative functional role in indicating immune relevance. So, we tried to identify the correlation between immune activity and risk score by deconvoluting the tumor immune microenvironment from the transcriptome (Fig. 1E), and showed that the immune cells and immune activity are indeed involved in tumor patients in the risk model.

### Dataset preparation and data processing

The results here are in part based from data generated by the TCGA Research Network: https://www.cancer.gov/tcga.

A cohort of 437 samples with clinical annotations and follow-up information were included in our study. Transcriptome profiling data (HTSeq-FPKM files downloaded from The Cancer Genome Atlas TCGA, https://portal.gdc.cancer.gov) were used as the main data set for risk model establishment and evaluation. All gene expression quantification files from the cohort were downloaded as txt format and further merged together in one file for downstream analysis.

The validation dataset of GSE39582 containing 566 samples from 562 patients was downloaded from the research work of Laetitia Marisa et al. [10].

Hallmark gene sets were downloaded from Yin He et al. [11] (https://www.ncbi.nlm.nih.gov/pmc/articles/PMC6310928/), which addressed immune functions in different detailed aspects.

CMS subtyping data and Kras/Braf mutation information of the TCGA-COAD dataset are from the research work of Justin Guinney et al. [12].

Manual curation data of treatment and response information in the TCGA-COAD dataset is from the work of Enrico Moiso [13].

The dataset containing both proteome and transcriptome data is from the project CPTAC-2 prospective and is downloaded from cBioPortal database [14].

All the datasets are summarized in Additional file 1: Table S1 and can be downloaded directly from the indicated websites. Datasets or custom scripts that are used in this research can be obtained upon request.

### Data visualization and statistical analysis

R software (version 3.5.1, http://www.r-pro-ject.org) was used to analyze data and plot graphs. Boxplot and point plot were generated with R package “ggplot2”, heatmap scaled by row was generated by R package “pheatmap” with a clustering distance of “euclidean”. Chord diagram was generated with R package ‘circlize’. DEG statistical analysis is performed by Wilcox test with R function “wilcox.test”. Welch’s t test (unpaired) or one-way analysis of variance was used to analyze differences between groups in variables with a normal distribution.

### Gene co-expression network construction

**Fig. 2:**
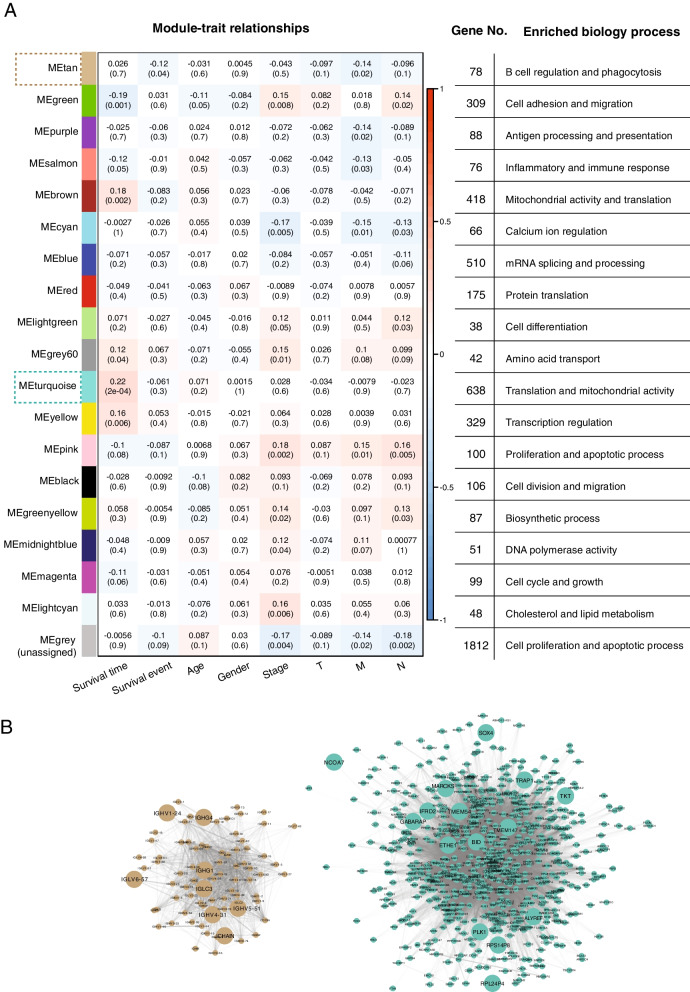
Module-trait relationships identified by WGCNA analysis from the transcriptome data of COAD patients. **A** Each colored module represents a network of genes with correlated expression built using TCGA-COAD samples after WGCNA quality filter and outliers’ removal (n = 283). “MEgrey” is unassigned gene sets which could not fit anywhere. Eight clinical traits (survival time, survival event, age, gender, stage, T, M, N) were evaluated for correlated gene expression networks. The correlation coefficients and p values are based on biweight midcorrelation. The correlation coefficients by modules and traits are shown at the top of each cell. The corresponding p-values for each module displayed at the bottom of each cell within parentheses. The rows are colored based on the correlation of the module with the indicating traits: red for positive and blue for negative correlation. Gene number and enriched biology process in each module of gene networks are displayed in the corresponding row on the right side. **B** Interaction network of module tan and module turquoise, adjacency threshold for including edges is 0.01 (nTan = 78; nTurquoise = 638). Connection strength between two genes is measured by the edge weight in the Topological Overlap Matrix, higher value refers to a stronger co-expression of genes, which is represented by the line transparency in the network. The network is organized by the force-directed layout, highly connected genes is centered in the network. The risk model genes (n = 23) is enlarged in the network

Co-expression networks were constructed by using WGCNA (v1.69) package [15, 16] in R. First of all, TCGA-COAD samples with clinical traits were selected after removing repetitions from the same patient (Additional file 1: Fig. S1A), then unqualified samples for WGCNA analysis are filtered by the function of “goodSamplesGenes”, ending up with 288 COAD samples. Uncommon genes and those with counts less than 10 in more than 90% samples are removed from the WGCNA analysis. After sample clustering, five outliers are detected and removed in the downstream module analysis with 283 remaining samples. A soft threshold (power) of 7 is chosen for network construction by function of “blockwiseModules” according to the scale-free topology criterion which referred to the smallest value for an approximate scale free topology as WGCNA used the topological overlap measure (TOM) to represent proximity (Additional file 1: Fig. S1B). Based on the topological overlap matrix measured from a pairwise correlation-based adjacency matrix, the neighborhood similarity among genes were estimated and the gene co-expression modules, which are distinguished with different colors, were then identified by average linkage hierarchical clustering. Using the Dynamic Hybrid Tree Cut algorithm and a minimum module size of 30 genes, a total of eighteen modules were identified. The correlation analysis of different modules is based on the module eigengenes (MEs) which represent the first principal component of the expression profiles in a given module (Additional file 1: Fig. S1C). Linear regression between MEs and clinical traits is applied to identify the module-trait associations (Fig. 2A). Modules are linked to traits by function of “bicorAndPvalue”. Data visualization is performed by the built-in functions in WGCNA package. The visualization of the network in module tan and turquoise is performed by the software Cytoscape (v3.7.1) based on the connection information of topological overlap matrix from WGCNA analysis with an edge weight threshold of 0.01, ending up with all the nodes (genes) in module tan (n = 78) and turquoise (n = 638) shown in the network.

### DEG identification

Significant differentially expressed genes (adjusted p < 0.05, |log2(Fold Change)|> 1) are identified by comparing tumor samples (n = 398) with normal samples (n = 39) or risk-high group (n = 167) with risk-low group (n = 167) in the TCGA cohort. p-value is calculated by unpaired Wilcoxon test, p-value is adjusted by FDR method. Log2 fold change is calculated by mean expression (FPKM) of tumor versus normal group.

### Prognosis analysis

By using the function ‘coxph’ in R package ‘survival’, a Cox proportional-hazards regression model was used to evaluate the significance of each parameter to overall survival, both survival time and state are considered as response parameters in this analysis.

### Risk model construction

Univariant Cox analysis was first performed to predict the prognosis-related DEGs from module MEturquoise and MEtan. Risk model was then established by applying stepwise regression (‘step’ function in R) to multivariant Cox model (‘coxph’ function in R) on 23 candidate prognosis-related genes in order to get an optimal simple model without compromising the model accuracy. The strategy used for stepwise regression is “sequential replacement”, which is a combination of forward and backward selections. It starts with no predictors, then sequentially add the most contributive predictors. After adding each new variable of candidate genes, remove any variables that no longer provide an improvement in the model fit. In this way, 23 risk genes were obtained in the risk model. By applying this method, a coefficient was predicted on each gene based on survival time and state of patients (Additional file 1: Fig. S2D). A risk value was then calculated by summing up the multiplication of gene expression level and its coefficient. Subsequently, we performed risk analysis to evaluate the risk model. Low- and high-risk group are stratified by the median risk score in the TCGA-COAD cohort (n = 334).

### Survival analysis

The Kaplan–Meier method was used to draw survival curves and the log-rank test was used to evaluate differences by using the function ‘coxph’ in R package ‘survival’. A Cox proportional-hazards regression model was used to evaluate the significance of each parameter to overall survival. Time-dependent receiver operating characteristic (tROC) analysis was performed to measure the predictive power by the R package ‘survivalROC’ [17] with the parameter ‘method = “KM”’, and the areas under the curve at different time points [AUC(t)] of all the variables were compared.

### Cox analysis on risk score and other clinical parameters

Univariant or Multivariant Cox proportional-hazards (Cox-PH) regression model was applied on the risk score (categorizing the patients in low-risk or high-risk) and other clinical parameters (age, gender, stage. T, M, N) using R package ‘survival’.

### Nomogram analysis

A nomogram and a calibration curve were calculated and plotted using R function (cph, Survival, calibrate and nomogram) in the R package ‘rms’ [18] with the parameters ‘lp = F, maxscale = 100, fun.at = c(0.99,0.9,0.8,0.6,0.4,0.2,0.1)’ for ‘nomogram’. Nomogram is a pictorial representation of a complex mathematical formula. In the nomogram of this study, all the clinical variables, such as age, gender, stage, TNM phage and risk score were used to represent a statistical prognostic model that predicts a probability of cancer death. Basically, nomo score coming from the nomogram analysis, is a comprehensive parameter for predicting cancer risk by integrating all the clinical variables that were put in. Nomo scores were extracted by the function ‘formula_lp’ and ‘points_cal’ in R package ‘nomogramFormula’.

### Gene ontology analysis

Gene ontology analysis is performed with differentially expressed genes in web application DAVID (https://david.ncifcrf.gov) [19, 20]. All gene ontologies enriched significantly (p < 0.05, fisher’s exact test) are shown in the dot plot.

### Immune cells deconvolution

CIBERSORTx (http://cibersortx.stanford.edu/) was implemented to deconvolute the composition of specific immune cells in the tumor microenvironment (TME) from the transcriptome data of TCGA cohort. Specifically, FPKM matrix of COAD patients is formatted as input file and uploaded to the web application of CIBERSORTx, then cell fractions are imputed in the module of “Impute Cell Fractions” with custom mode and default parameters (permutations for significance analysis is set to 100). The signature matrix file built in CIBERSORTx (LM22: 22 immune cell types) is used as the signature reference in this analysis.

### Immune activity deconvolution

Single-sample gene set enrichment (ssGSEA) analysis (R package ‘gsva’ with parameters “method = 'ssgsea', kcdf = 'Gaussian', abs.ranking = TRUE”) was utilized to identify clusters with different immune activity referring the immune-activity hallmarks from Yin He et al. [11] in four different immune aspects: immune cells, immune and cytolytic activity, antigen presentation pathway, and cytokine response. The enrichment scores of each hallmarks gene set were summed to generate the SIES (Sum of immune enrichment scores) for each sample.

‘Estimation of STromal and Immune cells in MAlignant Tumours using Expression data’ (ESTIMATE) is a method that uses gene expression signatures to infer the fraction of stromal and immune cells in tumour samples. Stromal, Immune and Tumor scores were obtained by using R package of ESTIMATE [21]. The stromal score captures the presence of stroma in tumor tissue, immune score represents the infiltration of immune cells in tumor tissue. ESTIMATE score of each patient = immune score of each patient + the corresponding stromal score. As the higher the ESTIMATE score is, the lower the content of tumor cells is in the given tumor microenvironment. The tumor score was calculated by the following equation: Tumor score = maximal value of ESTIMATE score in TCGA-COAD cohort—ESTIMATE score of each colon cancer patient.

### Correlation analysis between SIES and risk score

As outliers affect the correlation analysis a lot, that is especially serious in the risk model where extreme values exist, those outliers statistically away from normal distribution are filtered from TCGA-COAD cohort before correlation analysis. Maximum and minimum are tested and filtered in a stepwise way by the function of “grubbs.test” until all the remaining risk scores fits a normal distribution. We end up with 313 samples from 334 samples in TCGA-COAD for downstream correlation in which unpaired two tailed t test are applied.

### The comparison between our risk model with other prognosis models from different researches in the colon cancer

The performance comparison is performed between our risk model and other models from the researches of Huang et al. [22], Chen et al. [23], Sun et al. [24], and Liu et al. [25]. The comparison is based on the metrics of − log10(p-value) in the discovery and validation cohort respectively. p-value is calculated by the log rank test in the survival analysis. For those p values recorded already in the indicated cohorts in the corresponding researches, they were used directly without any change. For those p values not recorded in the corresponding researches, we calculated the p value in the indicated cohorts by two groups stratified according to the median value of gene expression or risk score as defined in the corresponding researches. For those models which indicated the gene expression in more than one gene, we used the gene which had the best prognosis in the discovery cohort in the comparison.

## Results

### Establishment of a module-based gene signature for prognosis

In order to build a novel prognostic risk model from cancer transcriptome data, we downloaded RNAseq-based transcriptome data of the TCGA-COAD cohort (398 tumor samples from 334 patients of COAD). Hierarchical and clustering analysis of gene expression data revealed 5 samples clustering apart from all the others (Additional file 1: Fig. S1A). After exclusion of these 5 outlier samples, we performed weighted gene co-expression network analysis (WGCNA) to screen modules of highly correlated genes associated with the clinical parameters [15]. After choosing the soft threshold (Additional file 1: Fig. S1B), the adjacency matrix describing the correlation strength of each pair of nodes based on Pearson’s correlation of module eigengenes (MEs) is transformed into topological overlap measure (TOM), which quantitatively represents the similarity in genes by comparing connection strength of two genes’ adjacency with other genes. Subsequently, 18 different gene co-expression modules are generated by conducting hierarchical clustering to classify genes with similar expression profiles based on TOM dissimilarity.

The functional modules were compared for the patient prognosis traits (survival and survival time) to identify prognosis-related gene signatures in a systematic way. Cluster 1 (MEtan, MEgreen, MEpurple and MEsalmon) showed a negative correlation with either survival event or survival time, while cluster 3 (MElightgreen, MEgrey60, MEturquoise and MEyellow) showed a positive correlation with survival time (Fig. 2A and Additional file 1: Fig. S1C). Clusters 1 and 3 are enriched in immune-associated pathways and translation/transcription respectively, that may indicate the extrinsic and intrinsic factor in affecting the survival of COAD patients. Interestingly, cluster 4 (MEpink, MEblack and MEgreenyellow), which is positively correlated with traditional clinical parameters (Stage, M, and N), is enriched in cell proliferation, migration and biosynthetic processes, suggesting the important and complex role of these cellular activities in tumor progression (Fig. 2A).

As module MEturquoise and MEtan showed the highest absolute correlation with survival time and event respectively (0.22 and − 0.12), we further established the risk model based on the genes in these two modules (module-derived genes). DEG (differentially expression genes) analysis comparing tumor and normal samples in the TCGA cohort identified 96 upregulated and 78 downregulated genes in cancer from the 716 genes of the MEturquoise and MEtan modules (Additional file 1: Fig. S2A). These DEGs are strongly enriched in innate immune response pathways, adaptive immune response (B cell regulation and immunoglobulin production), and phagocytosis process (Additional file 1: Fig. S2B). It suggested that the immune response occurred in tumor may induce a different survival on COAD patients. We then applied univariant Cox regression on the module-derived DEGs and ended up with 23 genes that are correlated with prognosis (p < 0.05). Most immunoglobulin genes (IGHG, IGHV, IGLC, IGLV), GABARAP, MARCKS, and SOX4 are identified with a hazard ratio (p-value < 0.05) of more than 1 in univariant Cox regression (Additional file 1: Fig. S2C). Stepwise multivariant Cox regression was applied to establish the risk model based on the 23 prognosis genes (Additional file 1: Fig. S2D). In the risk model, NCOA7 comes up with the biggest coefficient. A risk score was calculated in each sample by summing up the multiplication of gene expression level and its coefficient in the 23-genes risk model. Network construction showed immunoglobulin genes in the risk model are connected in module tan and the other 15 genes are connected in module turquoise with BID, TMEM147, ETHE1 and TMEM54 highly interacted in the center, while NCOA7 established only a small amount of connections in this module (Fig. 2B). Since the molecular classification of COAD had been intensively investigated and CMS (consensus molecular subtypes) is a comprehensive parameter representing different molecular signatures [12], we checked the risk score in different CNS groups in the TCGA dataset and found CMS4 group, which was known to represent a worse overall survival and relapse-free survival [12], had a significantly larger risk score than other subtypes (Additional file 1: Fig. S2E). CMS subtyping is consistent with the prognosis effect that the risk model represents. In addition, the risk score in the Kras-mutated group is larger than the Kras-non-mutated group while it is lower in the Braf-mutated group than the Braf-non-mutated group in the TCGA-COAD cohort (Additional file 1: Fig. S2F and S2G). We didn’t observe a significant correlation between the risk score and Kras expression (p = 0.29) or MSI (p = 0.14) in the TCGA-COAD cohort (n = 334, data not shown). Other than that, no significant correlation was observed between the risk score and nonsynonymous mutation counts (n = 318, p = 0.18, data not shown, nonsynonymous mutation counts are from cBioPortal) in the TCGA-COAD cohort. We also analyzed risk score in patients after chemotherapeutic treatment and we observed that the responders to the FLUOROURACIL + LEUCOVORIN + OXALIPLATIN treatment exhibited lower risk score than the non-responders (Additional file 1: Fig. S2H), which indicated the potential relevance of the risk model to clinical treatments.

### Risk score serves as a risk factor for overall survival in the cohort

**Fig. 3:**
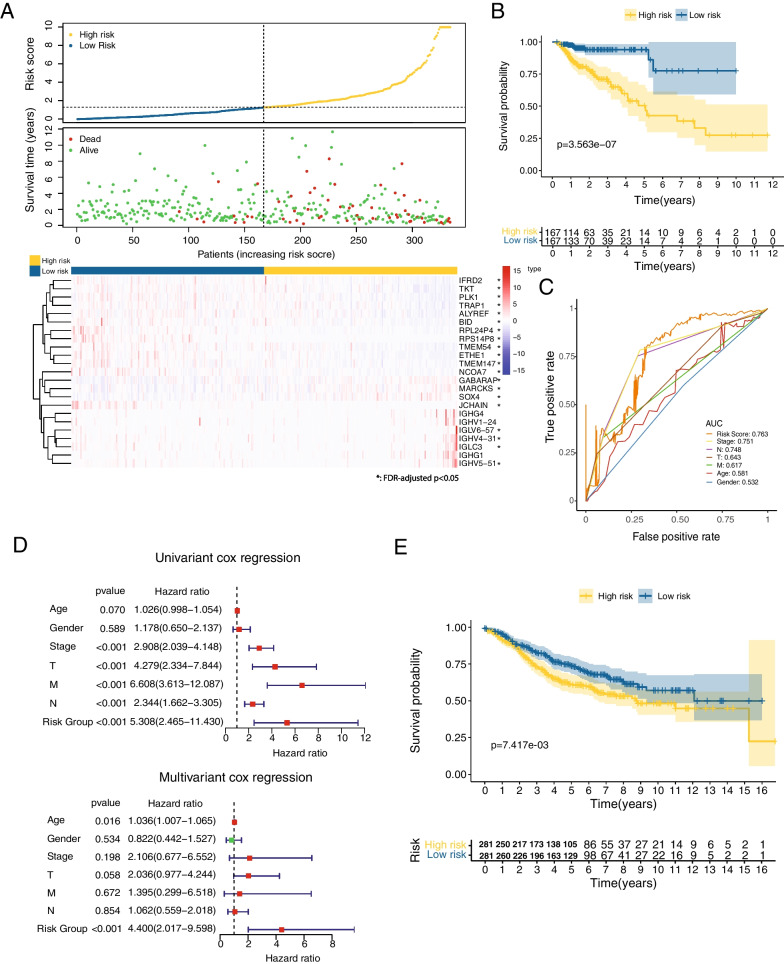
Survival and Hazard ratio analysis based on risk model genes. **A** Panels above: ranked patients by risk score and relative survival visualization. Panel below: heatmap of expression level of 23 risk model genes is shown in the indicated ranked patients. Risk groups are identified by the median risk score. *Differentially expressed between low-risk group and high-risk group (p-value is calculated by Wilcoxon test, FDR-adjusted p < 0.05). **B** Kaplan–Meier survival analysis in low- and high-risk group categorized by risk score in TCGA-COAD cohort (n = 334). **C** AUC (area under the ROC curve) of five-year survival predicted by risk score and all the other clinical parameters. TNM staging system is used to describe the amount and spread of cancer in a patient’s body. T: the size of the original (primary) tumor and whether it has invaded nearby tissue; N: spread of cancer to nearby lymph nodes; M: distant metastasis (spread of cancer from one part of the body to another). **D** Forest plot of Hazard ratios with 95% confidence intervals of the indicated risk factors is obtained with both univariant (above) and multivariant (below) Cox regression method. **E** Kaplan–Meier survival analysis in another independent cohort of colon cancer (GS39582E, n = 562)

To evaluate the risk score as a risk factor in prognosis, the COAD patients were divided into two equal parts as low-risk group and high-risk group according to the median risk score in the cohort and supporting the validity of the risk model, more dead cases were enriched in the high-risk group (Fig. 3A, upper panels). Of the 23 genes used to calculate the risk score, 20 genes were statistically differentially expressed between low- and high-risk groups with the majority of them being downregulated in high-risk group (Fig. 3A, bottom panel, genes with asterisks). Survival probability was significantly higher in low-risk group (p = 3.56e−07), with the probability of 5-year survival in low-risk group being 0.861 [95% CI 0.722–1] compared with 0.427 [95% CI 0.297–0.613] in high-risk group (Fig. 3B). AUC (area under the roc curve) of the risk model on predicting 5-year survival reaches 0.763 as the highest among all the clinical stratifications (Stage: 0.751, N-phase: 0.748, T-phase: 0.643, M-phase: 0.617, Age: 0.581, Gender: 0.532) (Fig. 3C). Both univariant (HR = 5.3, p < 0.001) and multivariant Cox analysis (HR 4.4, p < 0.001) showed the risk score could predict prognosis as an independent factor (Fig. 3D). Multivariant Cox regression modeling demonstrated that risk score and age are the only significant independent risk factors for overall survival among various clinicopathological variables (p < 0.05, Fig. 3D). In order to quantify the risk assessment for individual COAD patients, a nomogram was built by integrating the risk score to all the other clinicopathological features (Additional file 1: Fig. S3A). As expected, the nomo score was the most powerful and stable parameter in survival prediction across the whole-time course from 1 to 5 years (average AUC > 0.8). The nomo score 1 based on both our risk score and all the other clinical parameters was higher than the nomo score 2 which is only based on the clinical parameters, indicating our risk model can increase the prognosis effect of the traditional parameter system (Additional file 1: Fig. S3B). Risk score and stage exhibited similarly good prediction in the first 3-year survival (Additional file 1: Fig. S3B). Expression changes of the 23 risk genes grouped by clinicopathological features were summarized in Table S2 (Additional file 1). NCOA7 which showed decreased expression in dead cases, also exhibited decreased expression in later stage phased by stage, T, M, and N (Additional file 1: Fig. S3C–G). The correlation between NOCA7 and clinical phase may partially explain its biggest contribution in the risk model (Additional file 1: Fig. S2D). Furthermore, we checked the NCOA7 expression in the TCGA dataset and found NCOA7 increased expression in the tumor samples and low-risk group had a larger NCOA7 expression than high-risk group (Additional file 1: Fig. S3H). The difference of NCOA7 expression between low-risk group and high-risk group was also confirmed in the validated dataset (GSE39582; Additional file 1: Fig. S3I). Further validation of NCOA7 is performed by using its protein level (project CPTAC-2 prospective database), in agreement with the result obtained using the RNA expression, it showed a negative correlation between NCOA7 expression level and risk score (Pearson correlation, R = − 0.56, p = 1.9e−05; Additional file 1: Fig. S3J). Interestingly, NCOA7 (Nuclear Receptor Coactivator 7) has been reported to have gene polymorphisms associated with breast cancer development [26, 27] and it has also been identified as a potential biomarker in oral squamous cell carcinoma [28]. In addition, the engagement of NCOA7 by 3-HAA (3-hydroxyanthranilic acid) enhances the activation of AhR (aryl hydrocarbon receptor) in immunoregulatory dendritic cells [29].

To validate the prognosis effect of our risk model, survival analysis was performed in another independent cohort of colon cancer (GSE39582). Despite the fact that the study of GSE39582 employed a different methodology to profile the cancer transcriptome (Microarray instead of RNAseq), the survival probability was significantly increased in low-risk group with the probability of 5-year survival in low-risk group being 0.736 [95% CI 0.682–0.795] compared with 0.608 [95% CI 0.548–0.674] in high-risk group (Fig. 3E). These data strongly indicate that the risk score, obtained by using the identified 23 genes, has a robust prognosis value.

### Molecular signature of the risk model genes is represented in immune response

**Fig. 4:**
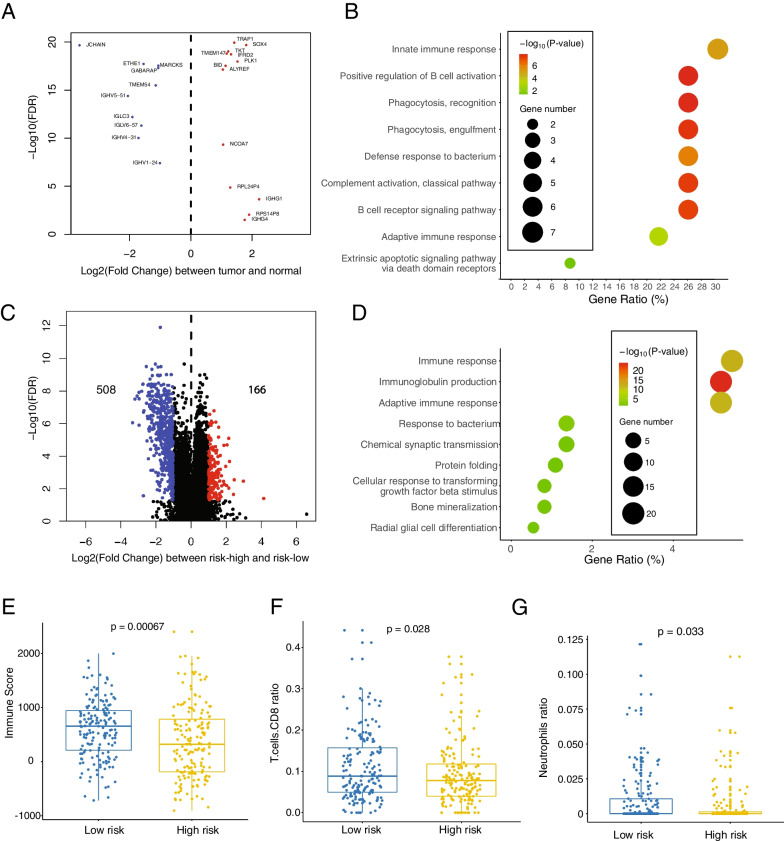
Functional representation of the 23 risk model genes revealed the relevance to the immune response. **A** Volcano plot showing the DEGs between tumor and normal samples in TCGA dataset (nCOAD = 334, nNormal = 39) regarding the risk model genes. **B** Gene ontology (GO) analysis of the 23 risk model genes. Significantly enriched GO terms (p < 0.05) are shown. Gene ratio = percentage of input genes involved in the pathway/percentage of all the pathway genes. p-value is calculated by fisher’s exact test. **C** Volcano plot showing the DEGs between high-risk group and low-risk group of COAD samples in TCGA dataset (nHigh-risk = 167, nLow-risk = 167), Significant DEGs is highlighted by adjusted p < 0.05 and |log2(fold change)|> 1. **D** Gene ontology (GO) analysis of the significant DEGs in **C**. Significantly enriched GO terms (p < 0.05) are shown. Gene ratio = percentage of input genes involved in the pathway / percentage of all the pathway genes. p-value is calculated by fisher’s exact test. **E** Boxplot indicating the immune score (calculated from ESTIMATE) in risk score-stratified groups (Low or High). p-value is calculated by unpaired two tailed t test. **F**, **G** Relative cell population ratio (estimated from CIBERSORTx) of the indicated immune cell types in risk score-stratified groups. p-value is calculated by unpaired two-tailed t test

To better understand the function of the 23 genes of the risk model, we compared tumor samples with normal samples; all the 23 genes were differentially expressed between normal and cancer tissue, with 10 genes downregulated and 13 upregulated (Fig. 4A). As expected, these genes were strongly enriched in innate immune response, B cell-related pathway, phagocytosis, adaptive immune pathway, and immunoglobulin components (Fig. 4B and Additional file 1: Fig. S4A). DEG analysis between the risk-high group and the risk-low group in the tumor samples identified 166 upregulated genes and 508 downregulated genes in the risk-high group (Fig. 4C). These DEGs are mainly enriched in “immune response”, “immunoglobulin production” and “adaptive immune response”, indicating a difference of the immune responses stratified by the risk model (Fig. 4D). To further characterize our risk model, we evaluated the tumor microenvironment (TME) enrichment by estimating the tumor, stromal and immune contribution to the sample's transcriptome by using the software ESTIMATE. Immune enrichment score was significantly higher in low-risk group patients, while no significant enrichments were observed for the ESTIMATE, the tumor and the stromal score (Fig. 4E and Additional file 1: Fig. S4B). Complementary analysis performed by using the software CIBERSORTx, aimed to estimate the cell populations ratio within the sample, showed a larger ratio of CD8 T cells and Neutrophils in low-risk group compared to high-risk group while no significant changes were observed for the other inferred cell populations (Fig. 4F and G). Taken together, these analyses indicated the relevance of the immune activity within the tumor for the performance of our risk model.

### Risk score was correlating with immunosuppressive TME in COAD

**Fig. 5:**
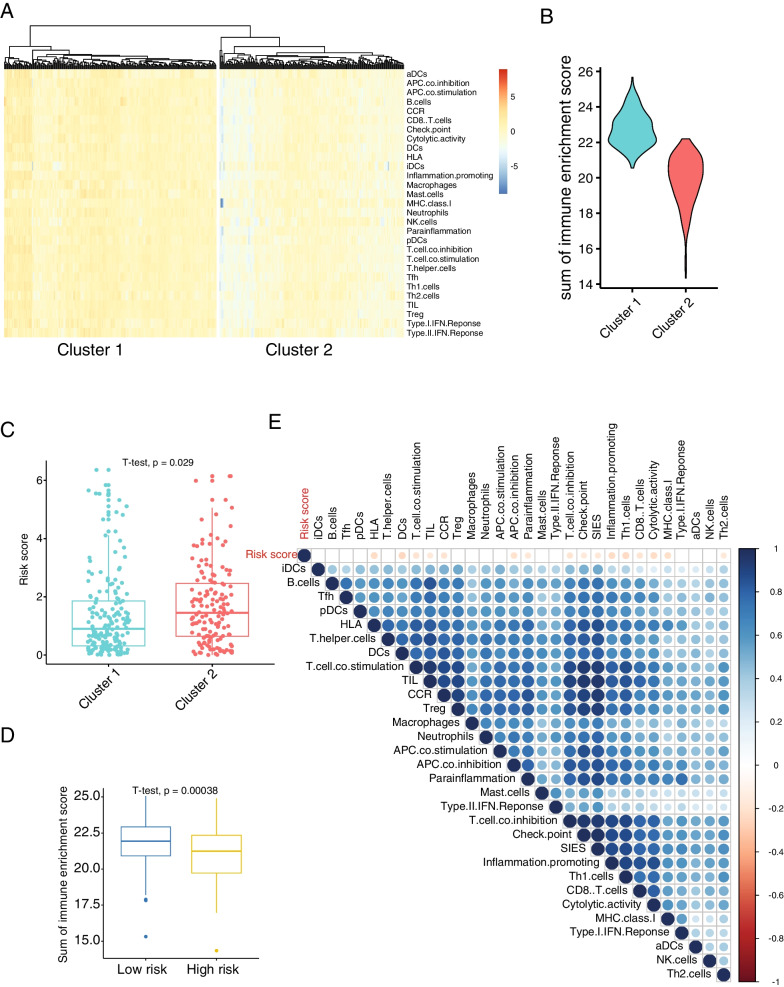
Risk score is correlating with immunosuppressive tumor microenvironment in COAD patients. **A** Hierarchical cluster and heatmap of the enrichment scores of the various gene sets related to immune functions on the COAD patients (n = 334). **B** Boxplot indicating the sum of the immune enrichment scores (SIES) in cluster1 and clsuter2. p-value is calculated by unpaired two tailed t test. **C** Boxplot indicating the risk score of COAD patients in cluster1 and cluster 2 (n = 313). p-value is calculated by unpaired two tailed t test. **D** Boxplot indicating the SIES in patients of low- and high- risk (according to the risk score stratification). p-value is calculated by unpaired two tailed t test. **E** Dot heatmap of the Pearson correlation between risk score and enrichment score of the various gene sets related to immune functions. Dot size indicate the correlation extent

To further investigate the role of risk score in the process of immune regulation as a prognosis factor and its association with the tumor immune environment, single-sample gene set enrichment analysis (ssGSEA) was executed on immune-associated gene sets. Enrichment scores of various gene sets related to immune system were calculated from transcriptomes of the COAD patients and hierarchical clustering together with heatmap visualization showed two distinct categories (Fig. 5A). Sum of immune enrichment scores (SIES, defined as the sum of the enrichment scores from all the immune-associated gene sets in Fig. 5A) is higher in cluster1 than in cluster2 (Fig. 5B).

To confirm the immune characteristics of these two clusters, we further assessed the SIES with the stromal score, immune score and tumor score inferred from the software ESTIMATE (that estimates the stromal/immune/tumor contribution to the tumor transcriptome). SIES was positively correlated with ESTIMATE score (r = 0.8), stromal score (r = 0.61) and immune score (r = 0.89), while negatively correlated with tumor score (r = − 0.8) (Additional file 1: Fig. S5A), suggesting that SIES correlates accurately with the immune activity and that a higher immune activity is present in cluster1 with respect to cluster2. Cluster1 exhibited a lower risk score than cluster2 (Fig. 5C) and the expression of immunoglobulin genes (IGHG, IGHV, IGLC, IGLV) are significantly decreased in cluster 2 (Additional file 1: Fig. S5B), indicating immunoglobulin expression can bona fide reflect the immune activity. Finally, COAD patients of risk score-inferred low-risk group have a higher SIES than those of high-risk group (Fig. 5D).

SIES consists of various immune aspects, which allow us to deconvolute the immune activity represented by SIES (Fig. 5E). In particular, gene sets related to dendritic cells, neutrophils and T cells (Th1 cells, T helper cells, TIL, CD8 T cells) and to a minor extent to some types of T-cells (e.g., Th2 cells, Treg, Tfh) are overrepresented in low-risk group (Fig. 5E and Additional file 1: Fig. S6). Not only immune cells but also immune cytolytic activity antigen presentation pathway and CCR response (Additional file 1: Fig. S6) were all increased in low-risk group, indicating a stronger innate (dendritic cells, neutrophils) and adaptive (T cells) immune response in the low-risk patients, which could partially explain the prognosis effect reflected by risk model.

## Discussion

It has been acknowledged that tumor microenvironment plays an important role in the development of colorectal cancer as an extrinsic factor [30]. The immune system has been found to be involved in both preventing and promoting tumor development [31]. It is known that there is wide crosstalk between epithelial cells and resident immune cells in colon through cytokines to maintain homeostasis and to coordinate appropriate responses to disease [32]. Understanding the immune processes involved in colorectal cancer helps us to establish novel markers for prognosis and expedite the progress of immune-based therapeutics [8]. The exploration of a robust immune-involved signature related to the prognosis of colorectal cancer would provide targets for treatment or other treatment paradigms for colorectal cancer. Some efforts on colon cancer had been made to explore prognosis-related signatures based on the TCGA dataset, most of them were based on the prior knowledge, resulting in a bias on the final selected gene sets [23–25, 33]. Other researches in this field were either focused on the relevance of individual genes’ expression [22, 23] or lacking robust validation and investigation [22, 23, 33], that could not exclude the possibility of an overfitting from the algorithm [33]. Furthermore, few established immune-associated signatures have been integrated with traditional prognostic systems in order to optimize the clinical routine.

In this study, we integrated the unbiased systematic analysis and clinical information to construct a comprehensive risk model related to prognosis in colorectal cancer. An immune-associated signature of 23 module-derived genes was selected in the risk model to generate a risk score by assigning different weights of each target gene. Prognosis analysis on the risk model suggested that risk score could provide an accurate risk stratification as an independent prognosis factor. Validation of the risk model on another large independent cohort of colon cancer proved that the indicated immune signature could work as robust integrated markers in COAD prognosis. As a way to improve the risk model, nomogram provided a more powerful decision than the traditional prognostic system. By comparing our risk model with other prognosis models from different researches of Huang et al. [22], Chen et al. [23], Sun et al. [24], and Liu et al. [25], no overlap is observed between our risk model with all the other models in the gene level, indicating the uniqueness of our risk model (Additional file 1: Fig. S7A). Our risk model also outperformed all the other models both in the discovery cohort and in the validation cohort in the survival analysis of colon cancer (Additional file 1: Fig. S7B).

Function representation analysis on the risk model indicated a large relevance between risk genes with immune response. Further investigation on the immune activity and its deconvolution identified the composition of immune cells and immune activity in tumor microenvironment (TME) of COAD patients, a low immune activity in the high-risk group may be responsible for the bad prognosis in patients with COAD. In summary, we identified a totally novel risk model, consisting of a comprehensive and new immune signature derived from systematic analysis. The risk model is proven to be robustly relevant for the prognosis of COAD patients in two (discovery and validation) independent cohorts. We also performed a complete investigation of the risk model and the immune activity.

Despite these promising results, more work needs to be done. Firstly, due to the limitations of retrospective studies, the prognostic robustness and clinical value of the risk model require further validation in larger, preferably prospective trials. Secondly, further experimental research could be undertaken to investigate the immune-associated biological functions underlying the risk genes in COAD, especially the immune mechanism of NOCA7 in COAD prognosis, which played a pivotal role in the risk model.

## Conclusion

In summary, we established a novel, validated immune-associated and module-derived gene signature to discriminate low-risk and high-risk patients with COAD in an unbiased way by applying systematic analysis. Integrating this with clinicopathological features, we constructed a nomogram to quantify risk assessment for individual patients. The robust immune gene signature-based model could be an effective tool to select high-risk patients who may benefit from targeting therapies and thus facilitate personalized management of COAD.

## Supplementary Information

**Additional file 1** Supplementary Information includes Abbreviations, 7 Supplementary Legends and Figures, 2 Supplementary Tables.

## Data Availability

The results shown here are in part based upon data generated by the TCGA Research Network: https://www.cancer.gov/tcga. All the datasets could be downloaded directly from the indicated websites. Datasets and custom scripts are available upon request.
