# Supplementary material for: Establishment and evaluation of module-based immune-associated gene signature to predict overall survival in patients of colon adenocarcinoma
Source: J Biomed Sci. 2022 Oct 14;29:81. doi: 10.1186/s12929-022-00867-2 (PMC9563160; doi:10.1186/s12929-022-00867-2)
Supplement: Supplementary file 1 — Additional file 1 Supplementary Information includes Abbreviations, 7 Supplementary Legends and Figures, 2 Supplementary Tables. [file 12929_2022_867_MOESM1_ESM.pdf]

Supplementary Files include Abbreviations, 7 Supplementary Figures, 2 Supplementary Tables.

Abbreviations
3-HAA: 3-hydroxyanthranilic acid
aDCs: Activated dendritic cells
AhR: aryl hydrocarbon receptor
APC: Antigen-presenting cell
AUC: Area under the ROC curve
CCR: Cytokine and cytokine receptor
Cox-PH: Cox proportional-hazards
DCs: Dendritic cells
DEG: Differentially expressed gene
FDR: False Discovery Rate
FPKM: Fragments per kilobase of exon per million mapped fragments
GSEA: Gene-set enrichment analysis
GSVA: Gene set variation analysis
HLA: Human leukocyte antigen
HR: Hazard ratio
HR.95L: The lower bounds of 95% confidence interval of HR
HR.95H: The upper bonds of 95% confidence interval of HR
iDCs: Immature dendritic cells
MEs: Module eigengenes
MHC: Major histocompatibility complex
NK.cells: Natural killer cells
OS: Overall survival
pDCs: Plasmacytoid dendritic cells
SIES: Sum of immune enrichment scores
ssGSEA: Single-sample gene set enrichment analysis
TCGA: The Cancer Genome Atlas
Tfh: Follicular helper T cells
TILs: Tumor-infiltrating lymphocytes
TME: Tumor microenvironment
TOM: Topological overlap measure
Treg: Regulatory T cells
WGCNA: weighted gene co-expression network analysis
CMS: consensus molecular subtypes

Supplementary Figures Legends

Figure S1. WGCNA analysis identified 18 modules of gene correlation networks. (A) Hierarchical clustering based on the transcriptomic data of the TCGA-COAD cohort (n=288) to detect outliers. (B) Scale-free network test by stepwise power in WGCNA analysis. The soft thresholding is a value used to power the correlation of the genes to that threshold. (C) Hierarchical clustering on the 18 identified modules by the Pearson correlation among module eigengenes.

Figure S2. A risk model is constructed based on module analysis and cox regression in COAD patients. (A) Volcano plot showing the DEGs between tumor and normal samples in TCGA dataset (nCOAD=334, nNormal=39) regarding the 716 genes from module MEturquoise and MEtan. (B) Gene ontology (GO) analysis of the DEGs from module ME MEturquoise and MEtan. Significantly enriched GO terms ($p<0.05$) are shown. Gene ratio = percentage of input genes involved in the pathway / percentage of all the pathway genes. P-value is calculated by fisher's exact test. (C) Forest plot of the Hazard ratios with 95% confidence intervals is obtained from univariant Cox regression analysis applied to DEGs from module MEturquoise and MEtan significantly correlated with prognosis ($p<0.01$). (D) The coefficient of each gene in the risk model. Coefficient: weight of each gene in the risk model; HR: hazard ratio; HR.95L: the lower bounds of 95% confidence interval of HR; HR.95H: the upper bonds of 95% confidence interval of HR. (E) Boxplot showing CMS4 subtypes (n=75) in TCGA-COAD dataset had a higher risk score than all the other consensus subtypes. P-value is calculated by unpaired two tailed t test. (F) Boxplot showing the Kras-mutated group (n=77) in TCGA-COAD dataset had a higher risk score than the non-mutated group (n=126). P-value is calculated by unpaired two tailed t test. (G) Boxplot showing the Braf-mutated group (n=19) in TCGA-COAD dataset had a lower risk score than the non-mutated group (n=184). P-value is calculated by unpaired two tailed t test. (H) Boxplot showing responders (n=25, including partial and complete response) of treatment of "FLUOROURACIL+LEUCOVORIN+OXALIPLATIN" had a lower risk score (p=0.017) than non-responders (n=5, including stable disease and clinical progressive disease). P-value is calculated by unpaired two tailed t test.

Figure S3. The prognosis effect of the risk model. (A) A nomogram is constructed to quantify risk assessment for individual patients. (B) AUC showing that nomo score which integrated risk score and other clinical parameters could improve the predicting accuracy on prognosis. "Nomo score 1" was based on all the parameters including risk score and other clinical parameters; "Nomo score 2" was based on only clinical parameters without considering risk score. (C, D, E, F, G) NCOA7 expression (FPKM) in groups of "alive cases" and "dead cases" (C), "stage I&II" and "stage III&IV" (D), "T1, T2, T3, T4" (E), "M0" and "M1" (F), "N0" and "N1-2" (G). (H) Boxplot showing in the TCGA dataset (transcriptome), tumor samples expressed more NCOA7 than normal samples, samples in the low-risk group expressed more NCOA7 than the high-risk group. P-value is calculated by unpaired two tailed t test. (I) Boxplot confirming more expression of NCOA7 in the low-risk group than the high-risk group in the tumor samples of the validation dataset of GSE39582 (transcriptome). P-value is calculated by unpaired two tailed t test. (J) Scatter plot showing the strong negative correlation between risk score and NCOA7 expression in the protein level (Pearson correlation R=-0.56, p=1.9e-05).

Figure S4. Molecular functions represented by the risk model genes. (A) Chord diagrams showing the functional enrichment represented by the risk model genes regarding biologyical process, molecular function, and cellular component. (B) Boxplot indicating the ESTIMATE/tumor/stromal score (calculated from ESTIMATE) in risk score-stratified groups (Low or High). ESTIMATE score of each patient = immune score of each patient + the corresponding stromal score. Tumor score = maximal value of ESTIMATE score in TCGA-COAD cohort – ESTIMATE score of each colon cancer patient. P-value is calculated by unpaired two tailed t test.

Figure S5. The expression and immune activity of 23 risk model genes are recapitulated by immune enrichment score. (A) Scatter plot showing the Pearson correlation (R) between SIES and ESTIMATE/immune/stromal/tumor score (calculated from ESTIMATE). ESTIMATE score of each patient = immune score of each patient + the corresponding stromal score. P-value is based on null hypothesis. Tumor score = maximal value of ESTIMATE score in TCGA-COAD cohort – ESTIMATE score of each colon cancer patient. (B) Expression heatmap of 23 risk model genes in COAD patients divided in cluster1 and cluster2 according to the enrichment scores as in Figure 5A. Risk score and stromal/immune/ESTIMATE/tumor score are also represented.

Figure S6. Immune activity in Low- and High- risk groups stratified by risk score. Boxplot showing the enrichment score distribution of various gene sets related to immune functions in risk score-stratified groups (Low or High). P-value is calculated by unpaired Wilcoxon test.

Figure S7. The comparison between our risk model with other prognosis models from different researches in the colon cancer. (A) Venn plot showing the gene sets from different prognosis models. (B) The comparison of the performance from different prognosis models in the discovery and validation cohort respectively. The gene expression level of MMP2 and MS4A4A is referred to stratify two groups for survival analysis in the research Huang et.al and Chen et.al respectively. P-value is calculated by the log rank test.

Figure S1

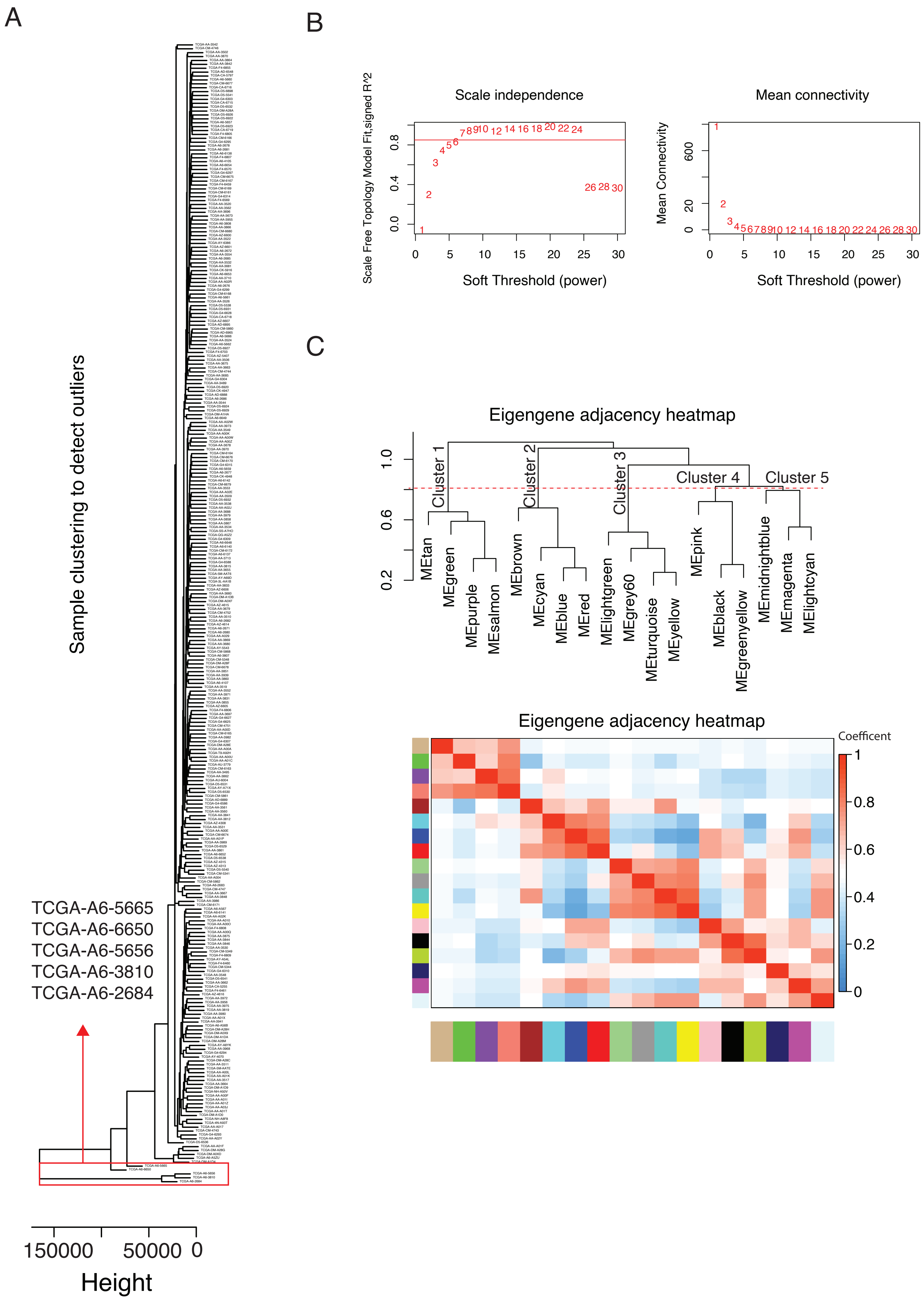

A
Sample clustering to detect outliers
TCGA-A6-5665
TCGA-A6-6650
TCGA-A6-5656
TCGA-A6-3810
TCGA-A6-2684
150000
50000
0
Height
B
Scale independence
Scale Free Topology Model Fit,signed R^2
Soft Threshold (power)
Mean connectivity
Mean Connectivity
Soft Threshold (power)
C
Eigengene adjacency heatmap
Cluster 1
Cluster 2
Cluster 3
Cluster 4
Cluster 5
MEtan
MEgreen
MEpurple
MEsalmon
MEbrown
MEcyan
MEblue
MEred
MElightgreen
MEgrey60
MEturquoise
MEyellow
MEpink
MEblack
MEgreenyellow
MEmidnightblue
MEmagenta
MElightcyan
Eigengene adjacency heatmap
Coefficent
1
0.8
0.6
0.4
0.2
0

Figure S2

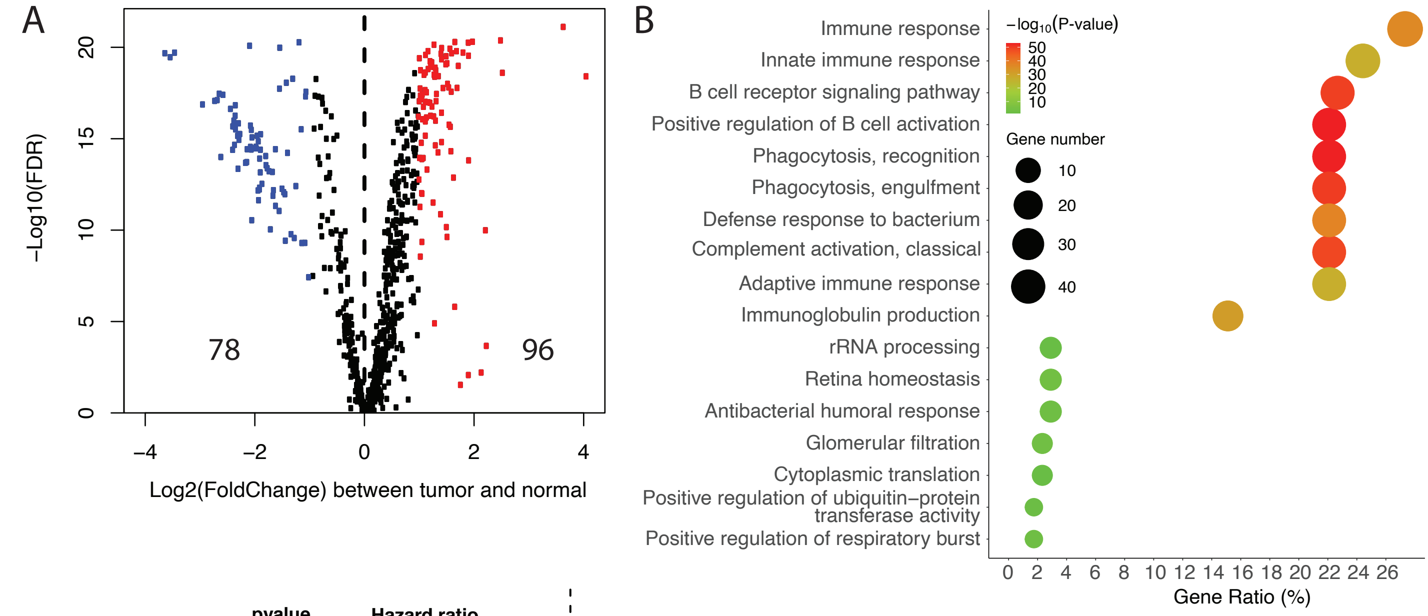

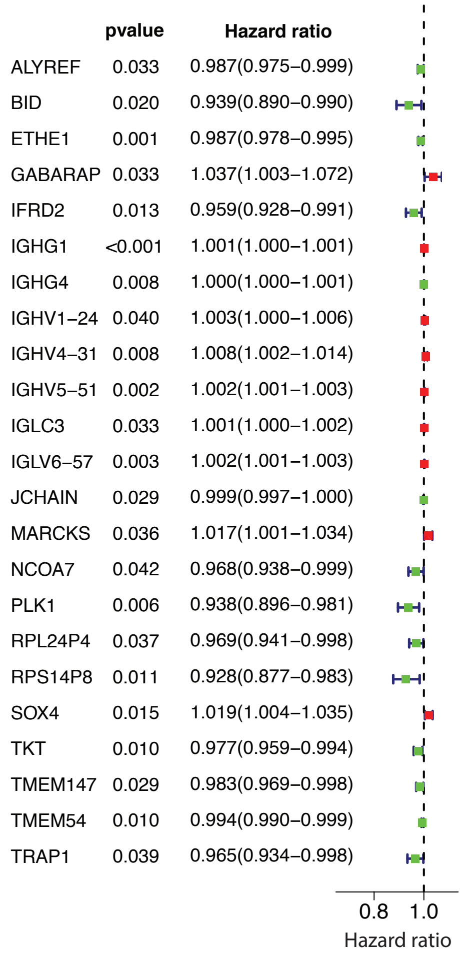

D

Gene	coef	HR	HR.95L	HR.95H
ALYREF	-0.00427	0.996	0.979	1.013
BID	0.01316	1.013	0.944	1.088
ETHE1	-0.00534	0.995	0.984	1.006
GABARAP	0.00067	1.001	0.953	1.051
IFRD2	0.01272	1.013	0.961	1.067
IGHG1	0.00003	1.000	0.999	1.001
IGHG4	0.00006	1.000	0.999	1.001
IGHV1-24	-0.00206	0.998	0.990	1.006
IGHV4-31	0.00999	1.010	0.994	1.026
IGHV5-51	-0.00049	1.000	0.997	1.002
IGLC3	0.00168	1.002	0.999	1.004
IGLV6-57	-0.00194	0.998	0.994	1.002
JCHAIN	-0.00251	0.997	0.995	1.000
MARCKS	0.01513	1.015	0.992	1.039
NCOA7	-0.0562	0.945	0.909	0.983
PLK1	-0.04015	0.961	0.895	1.031
RPL24P4	-0.02909	0.971	0.931	1.013
RPS14P8	-0.02704	0.973	0.912	1.038
SOX4	0.00239	1.002	0.980	1.025
TKT	-0.0116	0.988	0.962	1.016
TMEM147	0.00714	1.007	0.983	1.032
TMEM54	-0.00144	0.999	0.993	1.004
TRAP1	-0.00492	0.995	0.949	1.043

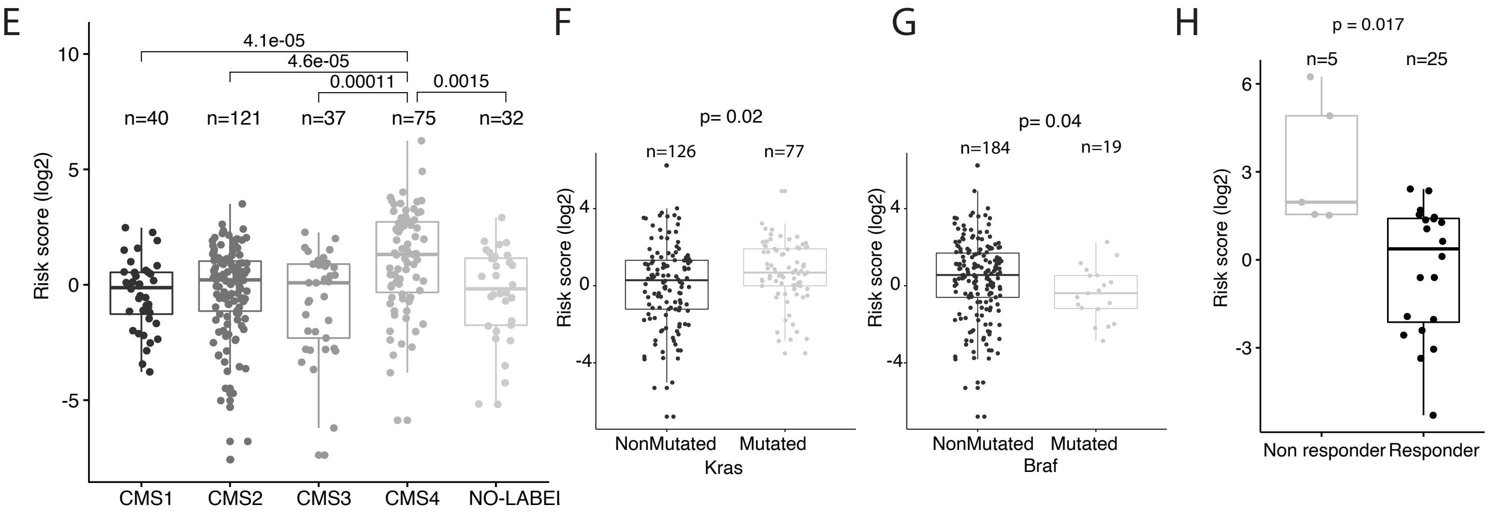

Figure S3

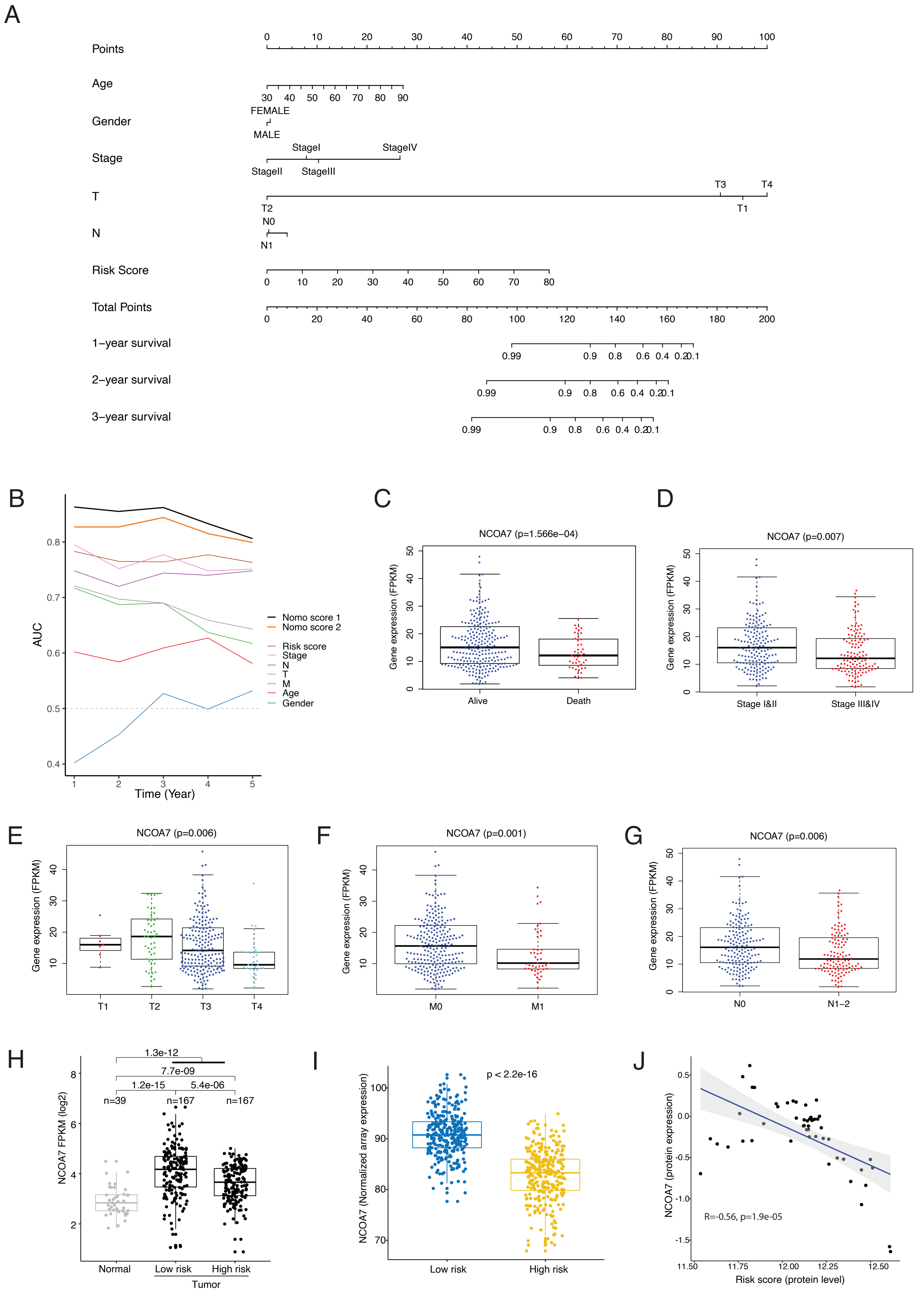
A
Points
Age
Gender
FEMALE
MALE
Stage
StageI
StageIV
StageII
StageIII
T
T3
T4
T2
T1
N
N0
N1
Risk Score
Total Points
1−year survival
2−year survival
3−year survival
B
AUC
Time (Year)
Nomo score 1
Nomo score 2
Risk score
Stage
N
T
M
Age
Gender
C
NCOA7 (p=1.566e−04)
Gene expression (FPKM)
Alive
Death
D
NCOA7 (p=0.007)
Stage I&II
Stage III&IV
E
NCOA7 (p=0.006)
T1
T2
T3
T4
F
NCOA7 (p=0.001)
M0
M1
G
NCOA7 (p=0.006)
N0
N1−2
H
NCOA7 FPKM (log2)
1.3e-12
7.7e-09
1.2e-15
5.4e-06
n=39
n=167
n=167
Normal
Low risk
High risk
Tumor
I
NCOA7 (Normalized array expression)
p < 2.2e-16
Low risk
High risk
J
NCOA7 (protein expression)
R=-0.56, p=1.9e-05
Risk score (protein level)

Figure S4

A

Biological process

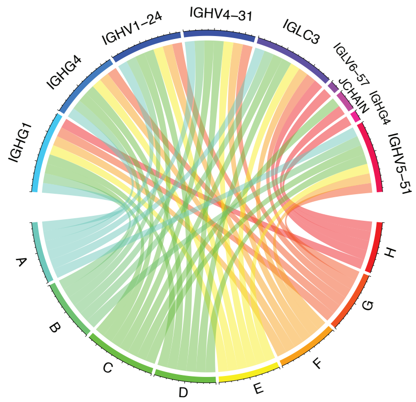

A: Positive regulation of B cell activation
B: B cell receptor signaling pathway
C: Innate immune response
D: Defense response to bacterium
E: Complement activation, classical pathway
F: Phagocytosis, engulfment
G: Phagocytosis, recognition
H: Adaptive immune response

Molecular function

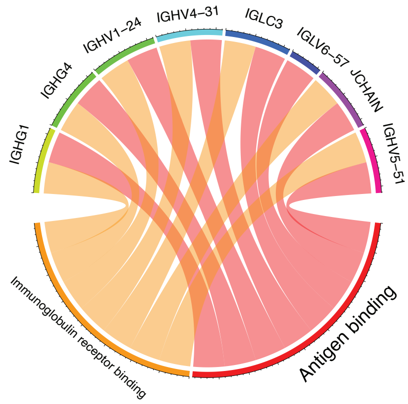

Cellular component

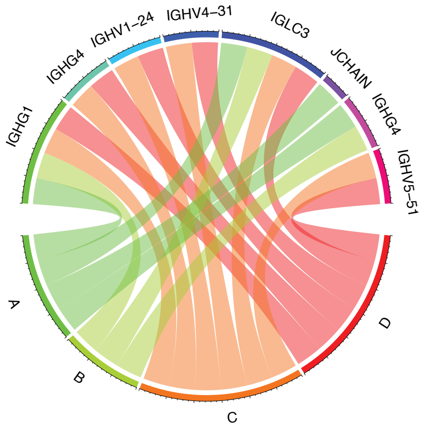

A: Blood microparticle
B: IgG immunoglobulin complex
C: Immunoglobulin complex, circulating
D: External side of plasma membrane

B

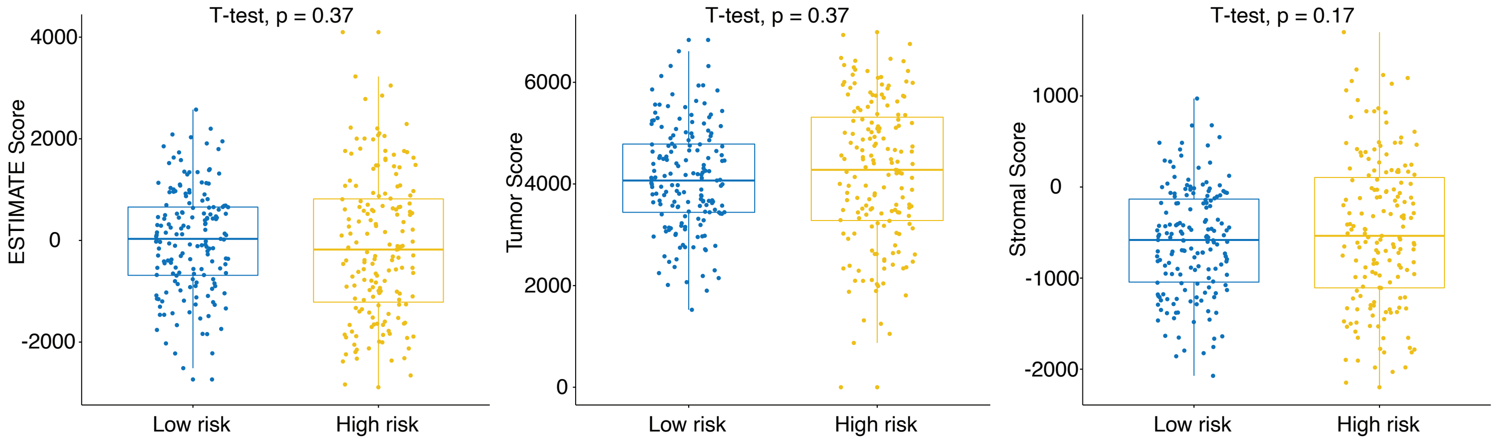

Figure S5

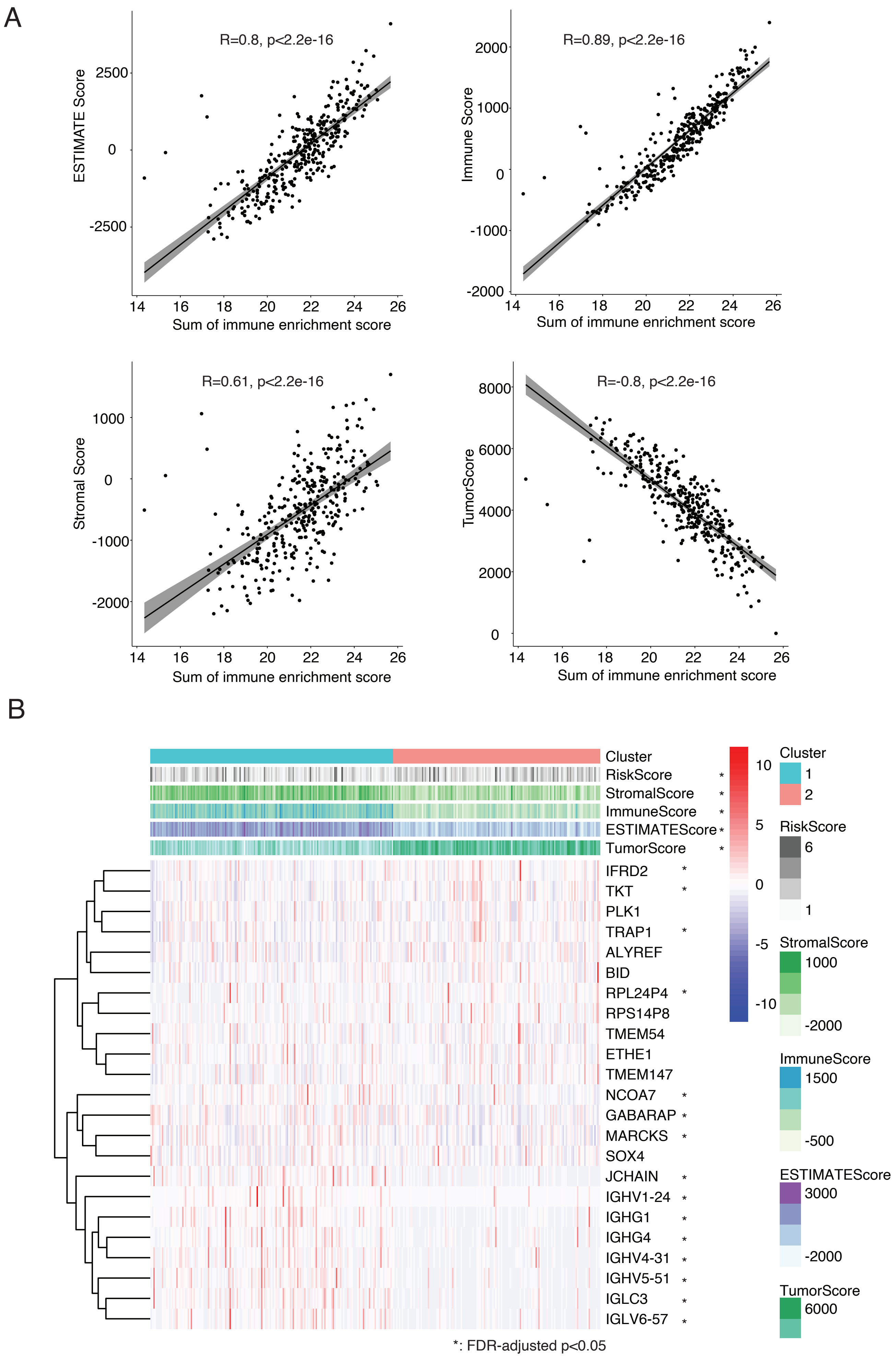
A
R=0.8, p<2.2e-16
ESTIMATE Score
Sum of immune enrichment score
R=0.89, p<2.2e-16
Immune Score
Sum of immune enrichment score
R=0.61, p<2.2e-16
Stromal Score
Sum of immune enrichment score
R=-0.8, p<2.2e-16
TumorScore
Sum of immune enrichment score
B
Cluster
RiskScore *
StromalScore *
ImmuneScore *
ESTIMATEScore *
TumorScore *
IFRD2 *
TKT *
PLK1
TRAP1 *
ALYREF
BID
RPL24P4 *
RPS14P8
TMEM54
ETHE1
TMEM147
NCOA7 *
GABARAP *
MARCKS *
SOX4
JCHAIN *
IGHV1-24 *
IGHG1 *
IGHG4 *
IGHV4-31 *
IGHV5-51 *
IGLC3 *
IGLV6-57 *
Cluster
1
2
RiskScore
6
1
StromalScore
1000
-2000
ImmuneScore
1500
-500
ESTIMATEScore
3000
-2000
TumorScore
6000
*: FDR-adjusted p<0.05

Figure S6

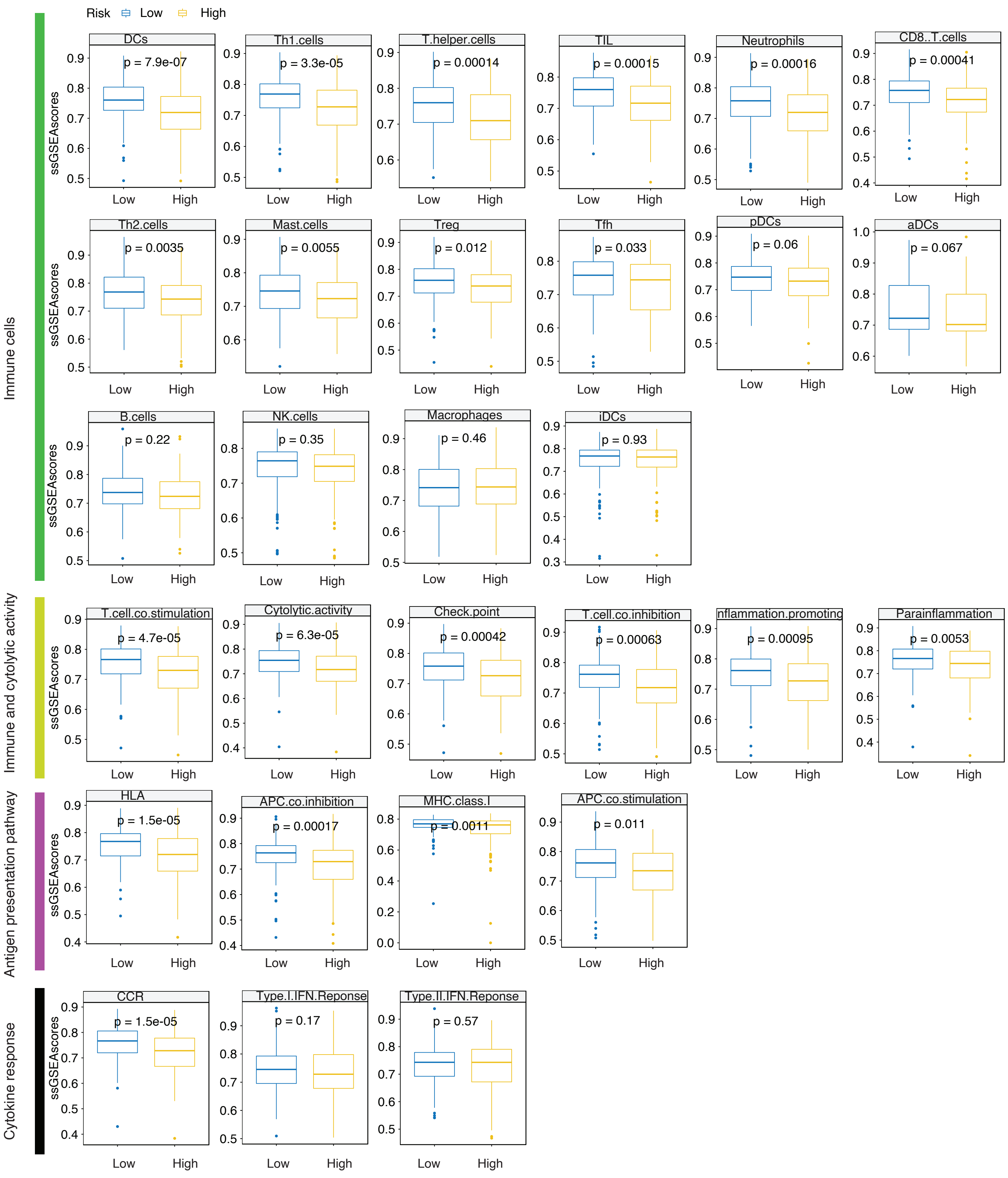

Risk Low High
Immune cells
Immune and cytolytic activity
Antigen presentation pathway
Cytokine response
ssGSEAscores
DCs p = 7.9e-07
Th1.cells p = 3.3e-05
T.helper.cells p = 0.00014
TIL p = 0.00015
Neutrophils p = 0.00016
CD8..T.cells p = 0.00041
Th2.cells p = 0.0035
Mast.cells p = 0.0055
Treg p = 0.012
Tfh p = 0.033
pDCs p = 0.06
aDCs p = 0.067
B.cells p = 0.22
NK.cells p = 0.35
Macrophages p = 0.46
iDCs p = 0.93
T.cell.co.stimulation p = 4.7e-05
Cytolytic.activity p = 6.3e-05
Check.point p = 0.00042
T.cell.co.inhibition p = 0.00063
nflammation.promoting p = 0.00095
Parainflammation p = 0.0053
HLA p = 1.5e-05
APC.co.inhibition p = 0.00017
MHC.class.I p = 0.0011
APC.co.stimulation p = 0.011
CCR p = 1.5e-05
Type.I.IFN.Reponse p = 0.17
Type.II.IFN.Reponse p = 0.57
Low High

Figure S7

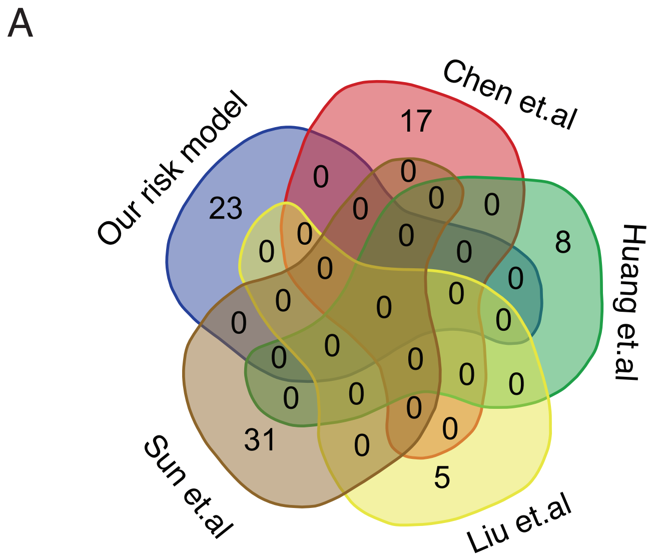
A
Our risk model
Chen et.al
Huang et.al
Sun et.al
Liu et.al
23
17
8
31
5

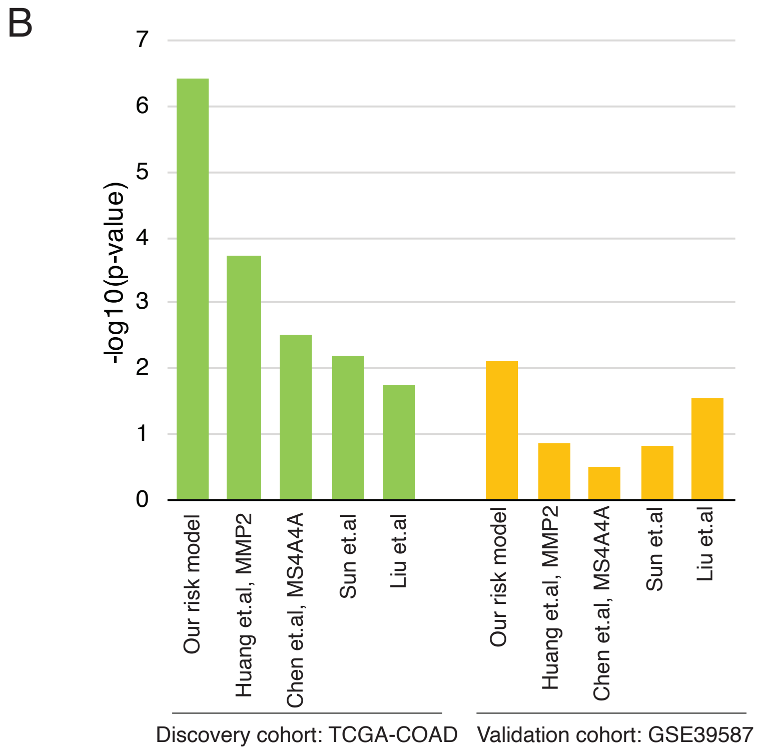
B
-log10(p-value)
0
1
2
3
4
5
6
7
Our risk model
Huang et.al, MMP2
Chen et.al, MS4A4A
Sun et.al
Liu et.al
Discovery cohort: TCGA-COAD
Validation cohort: GSE39587

Supplementary Tables

Table S1. Summarized datasets used in this study.

Data type	Source	Filtering	Statistics	Website	Download Date
Transcriptome profiling (FPKM)	TCGA-COAD	adenomas and adenocarcinomas	398 tumor samples from 334 patients and 39 normal samples	https://portal.gdc.cancer.gov/cart	2021.02
Clinical information	TCGA-COAD	adenomas and adenocarcinomas	398 tumor and 39 normal, filtered out samples whose survival time less than 90 days	https://portal.gdc.cancer.gov/cart	2021.02
Expression profiling by array	GSE39582	A large series of colon cancers, Affymetrix Human Genome U133 Plus 2.0 Array	566 samples from 562 patients of colon cancer	https://www.ncbi.nlm.nih.gov/geo/query/acc.cgi?acc=GSE39582	2022.06
Immune cell ratio	CIBERSORTx	TCGA samples	22 immune cell types	https://cibersortx.standford.edu/	2021.03
Hallmark geneset	Yin He et al.	13046_2018_1002_MOESM1_ESM.xlsx	29 immune signatures represented by 29 different gene sets	https://www.ncbi.nlm.nih.gov/pmc/articles/PMC6310928/	2018.12
Meta data containing CMS and other molecular classifications	Justin Guinney et al.	Annotated samples of colorectal cancer	573 annotated TCGA samples, 305 of which are shared in our risk model	https://www.synapse.org/#!Synapse:syn2623706	2022.08
Clinical data with manual curation of TCGA treatment information	Enrico Moiso	Manually curated by Enrico Moiso	33 TCGA-COAD samples with the treatment of "FLUOROURACIL+LEUCOVORIN+OXALIPLATIN", 30 of which are shared in our risk model	https://www.medrxiv.org/content/10.1101/2021.04.30.21251941v2.supplementary-material	2022.08
Protein and transcriptome data for the validation of NCOA7	Project CPTAC-2 Prospective from cBioPortal	Independent samples with protein data by TMT10 MS sequencing	105 samples, 51 of which detected protein expression of NCOA7 gene	https://www.cbioportal.org/study/clinicalData?id=coad_cptac_2019	2022.08

Table S2. Expression changes of the 23 risk model genes and risk score grouped by clinicopathological features.

Gene	Age (<=65 VS >65)	Gender (Male VS Female)	Stage (I&II VS III&IV)	T (1&2 VS 3&4)	M (0 VS 1)	N (0 VS 1&2)
ALYREF	-0.26(0.79)	-0.16(0.87)	1.68(0.10)	-0.03(0.98)	2.28(0.03)	1.77(0.08)
BID	-0.34(0.73)	0.19(0.85)	0.78(0.44)	2.31(0.02)	1.38(0.17)	0.75(0.45)
ETHE1	-0.16(0.87)	-0.45(0.65)	1.36(0.17)	-0.29(0.78)	0.69(0.49)	1.62(0.11)
GABARAP	1.61(0.11)	-0.37(0.71)	0.54(0.59)	-1.17(0.25)	-0.56(0.58)	0.28(0.78)
IFRD2	0.09(0.93)	0.35(0.72)	1.04(0.30)	1.64(0.11)	0.79(0.43)	1.51(0.13)
IGHG1	1.08(0.28)	0.27(0.79)	0.40(0.69)	-0.40(0.69)	0.41(0.68)	0.15(0.88)
IGHG4	0.09(0.93)	-1.80(0.07)	1.29(0.20)	0.54(0.59)	1.53(0.13)	1.12(0.27)
IGHV1-24	0.89(0.37)	-0.91(0.36)	0.26(0.80)	-0.22(0.83)	0.65(0.52)	0.09(0.93)
IGHV4-31	-0.87(0.39)	-1.80(0.07)	0.62(0.54)	0.99(0.33)	2.35(0.02)	0.44(0.66)
IGHV5-51	-0.04(0.97)	-1.81(0.07)	0.73(0.47)	0.12(0.91)	1.76(0.08)	0.55(0.58)
IGLC3	-0.30(0.76)	-1.40(0.16)	0.05(0.96)	-0.19(0.85)	1.86(0.07)	-0.05(0.96)
IGLV6-57	-0.48(0.64)	-0.85(0.40)	-0.47(0.64)	-0.01(0.99)	1.67(0.10)	-0.56(0.58)
JCHAIN	-1.00(0.32)	-1.94(0.05)	1.43(0.15)	0.23(0.82)	2.35(0.02)	1.62(0.11)
MARCKS	0.78(0.44)	-0.27(0.79)	-0.68(0.50)	0.74(0.46)	-0.34(0.74)	-1.23(0.22)
NCOA7	-0.30(0.76)	1.14(0.25)	2.73(0.01)	1.76(0.08)	3.36(0.001)	2.76(0.01)
PLK1	-0.10(0.92)	0.15(0.88)	-0.18(0.85)	0.16(0.88)	0.22(0.83)	0.19(0.85)
RPL24P4	-3.05(0.003)	-1.11(0.27)	0.2(0.84)	0.01(0.99)	1.57(0.12)	0.05(0.96)
RPS14P8	-1.10(0.05)	-1.81(0.07)	-0.27(0.79)	0.40(0.69)	1.39(0.17)	-0.08(0.94)
SOX4	1.21(0.23)	0.49(0.62)	-0.11(0.91)	0.41(0.68)	0.55(0.59)	-0.60(0.55)
TKT	-0.43(0.67)	0.82(0.41)	1.34(0.18)	1.77(0.08)	1.67(0.10)	1.31(0.19)
TMEM147	-1.46(0.15)	0.02(0.99)	1.20(0.23)	0.76(0.45)	0.58(0.57)	1.29(0.20)
TMEM54	-0.26(0.79)	0.12(0.91)	-0.09(0.93)	0.029(0.98)	-0.16(0.87)	0.57(0.57)
TRAP1	-0.47(0.64)	1.57(0.12)	0.87(0.39)	0.98(0.33)	-0.19(0.85)	1.44(0.15)
Risk score	0.29(0.77)	-0.86(0.39)	-1.67(0.10)	-2.04(0.04)	-0.96(0.34)	-1.78(0.08)
